# Efficacy of Miniscrew-Assisted Rapid Palatal Expansion (MARPE) in late adolescents and adults with the Dutch Maxillary Expansion Device: a prospective clinical cohort study

**DOI:** 10.1007/s00784-022-04577-9

**Published:** 2022-06-22

**Authors:** Aldin Kapetanović, Bieke M. M. J. Odrosslij, Frank Baan, Stefaan J. Bergé, René R. M. Noverraz, Jan G. J. H. Schols, Tong Xi

**Affiliations:** 1 Department of Dentistry - Orthodontics and Craniofacial Biology, Radboud Institute for Health Sciences, Radboud University Medical Center, Radboudumc, Dentistry 309, PO Box 9101, 6500 HB Nijmegen, the Netherlands; 2 Private Orthodontic Practice, Rotterdam, the Netherlands; 3 Radboudumc 3D Lab, Radboud Institute for Health Sciences, Radboud University Medical Center, PO Box 9101, 6500 HB Nijmegen, the Netherlands; 4 Department of Oral and Maxillofacial Surgery, Radboud Institute for Health Sciences, Radboud University Medical Center, PO Box 9101, 6500 HB Nijmegen, the Netherlands

**Keywords:** Miniscrew-Assisted Rapid Palatal Expansion (MARPE), Transverse maxillary deficiency, Midpalatal suture expansion, Maxillary expansion technique, Non-surgical expansion technique, CBCT

## Abstract

**Objectives:**

To provide a higher degree of evidence on the efficacy of Miniscrew-Assisted Rapid Palatal Expansion (MARPE) in late adolescents and adults, thereby applying the Dutch Maxillary Expansion Device (D-MED).

**Materials and methods:**

D-MED was developed as an individualized, 3D-designed, and fabricated MARPE appliance supported by 4 palatal miniscrews. Patients from the age of 16 onwards with transverse maxillary deficiency were enrolled consecutively. Pre-expansion and immediate post-expansion CBCTs and intra-oral scans were acquired and measurements of skeletal, alveolar, and dental expansion as well as dental and periodontal side-effects were performed.

**Results:**

Thirty-four patients were enrolled (8 men, 26 women) with mean age 27.0 ± 9.4 years. A success rate of 94.1% was achieved (32/34 patients). The mean expansion duration, or mean observation time, was 31.7 ± 8.0 days. The mean expansion at the maxillary first molars (M1) and first premolars (P1) was 6.56 ± 1.70 mm and 4.19 ± 1.29 mm, respectively. The expansion was 60.4 ± 20.1% skeletal, 8.1 ± 27.6% alveolar, and 31.6 ± 20.1% dental at M1 and 92.2 ± 14.5% skeletal, 0.0 ± 18.6% alveolar, and 7.8 ± 17.7% dental at P1, which was both statistically (*p* < 0.001) and clinically significant. Buccal dental tipping (3.88 ± 3.92° M1; 2.29 ± 3.89° P1), clinical crown height increase (0.12 ± 0.31 mm M1; 0.04 ± 0.22 mm P1), and buccal bone thinning (− 0.31 ± 0.49 mm M1; − 0.01 ± 0.45 mm P1) were observed, while root resorption could not be evaluated.

**Conclusions:**

MARPE by application of D-MED manifested its efficacy in a prospective clinical setting, delivering a high amount of skeletal expansion with limited side-effects in late adolescents and adults.

**Clinical relevance:**

Higher quality evidence is supportive of MARPE as a safe and successful non-surgical treatment option for transverse maxillary deficiency.

## Introduction

Miniscrew-Assisted Rapid Palatal Expansion (MARPE) has recently gained international attention as a non-surgical treatment for transverse maxillary deficiency in late adolescents and adults. Conventional Rapid Palatal Expansion (RPE) is a well-documented treatment for maxillary expansion in growing patients [1]. In late adolescents and adults, however, the skeletal effect of RPE was limited and it resulted in significant dental side-effects. Surgically-Assisted Rapid Palatal Expansion (SARPE) was, hence, considered necessary to enable separation of the midpalatal suture, but it was associated with the inherent surgical risks, higher costs, and comorbidity of a surgical procedure [2, 3]. A less invasive approach to expand the maxilla was thus envisaged.

Previous histological and radiological studies reported that the ossification of the midpalatal suture displayed a great variation and was often incomplete in late adolescents and adults, providing the basis for the development of MARPE [4]. Lee et al. were among the first to design a MARPE appliance as a tooth-bone-borne RPE-hyrax device anchored in the palate with four miniscrews through helical loops, which were soldered to the expansion screw base. The miniscrews were instrumental for transferring the expansion force directly into the palate, leading to separation of the midpalatal suture [5].

Moon et al. enhanced the MARPE appliance and introduced the Maxillary Skeletal Expander (MSE), which was positioned posteriorly, at the level of the first molars, in order to overcome the high skeletal resistance in this area, resulting in a more parallel suture expansion [6]. As the MSE was based on a prefabricated expansion screw and connectors that had to be soldered to the molar bands by the dental technician, a weakness in the metal structure was introduced that was more prone to deformation and fracture [7]. The shortcomings of the available expanders inspired the development of the Dutch Maxillary Expansion Device (D-MED), which was used in this study.

Several publications have examined the efficacy of MARPE, including a recent systematic review and meta-analysis. The findings suggested that MARPE was an efficient treatment for maxillary expansion in late adolescents and adults, but also that the vast majority of included studies was of retrospective nature and had a high risk of bias, especially regarding patient selection, and indicated the need for prospective studies of higher quality [8].

The aim of the present prospective clinical cohort study was to provide a higher degree of evidence on the efficacy of MARPE in late adolescents and adults, thereby applying the Dutch Maxillary Expansion Device. The primary objective was to assess success rate and skeletal and dentoalveolar treatment effects. The secondary objective was to assess potential dental and periodontal side-effects.

## Materials and methods

A prospective clinical cohort study was set up in October 2018. The study was approved by the Radboud University Medical Center Institutional Review Board (IRB no. 2019–5898) and a written informed consent was obtained from all participants. The study was performed in accordance with the Declaration of Helsinki. All data were anonymized and de-identified prior to analyses.

The inclusion criteria were consecutive patients from the age of 16 onwards who presented with transverse maxillary discrepancy (unilateral, bilateral, anticipated, or constriction without crossbite). The exclusion criteria were a history of maxillofacial surgery, cleft lip and palate, craniofacial anomalies or syndromes, congenital tooth anomalies, absent first or second molars, or extensive prosthetic restorations in the molar region. The skeletal maturation stage of the included patients was evaluated with the cervical vertebral maturation method on lateral cephalograms [9].

### Appliance design

**Fig. 1:**
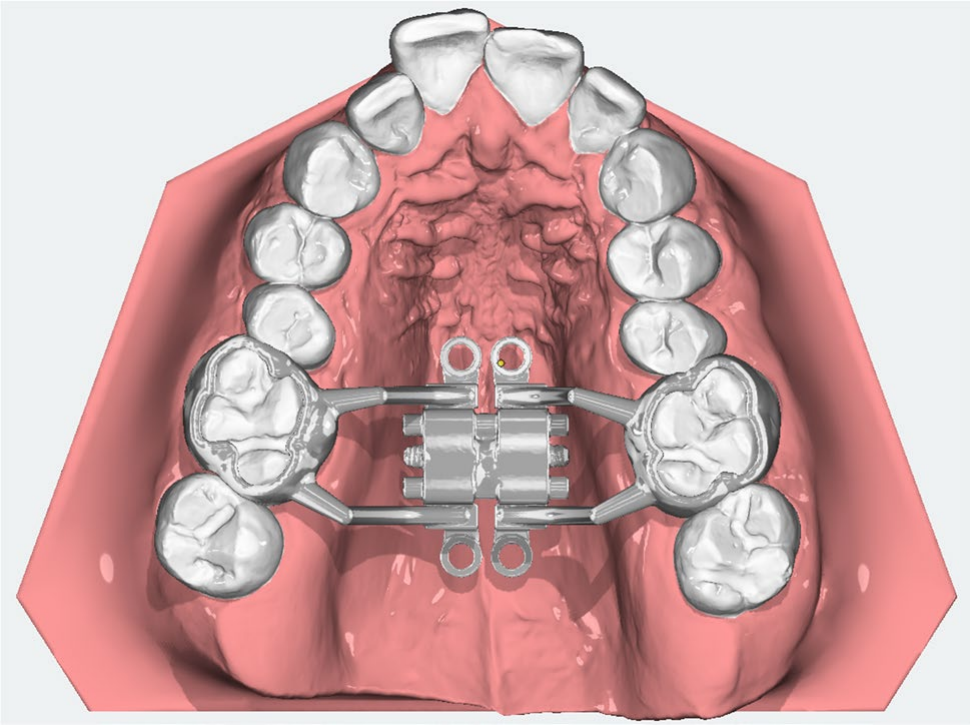
Occlusal view of the digital D-MED design on intra-oral scan

A fully digitally designed MARPE appliance was developed (see: Fig. 1) in a joint project by the Departments of Orthodontics and Oral & Maxillofacial Surgery with the intention of overcoming the design flaws of the existing MARPEs, such as the modified hyrax-type MARPE expander and the MSE.

**Fig. 2:**
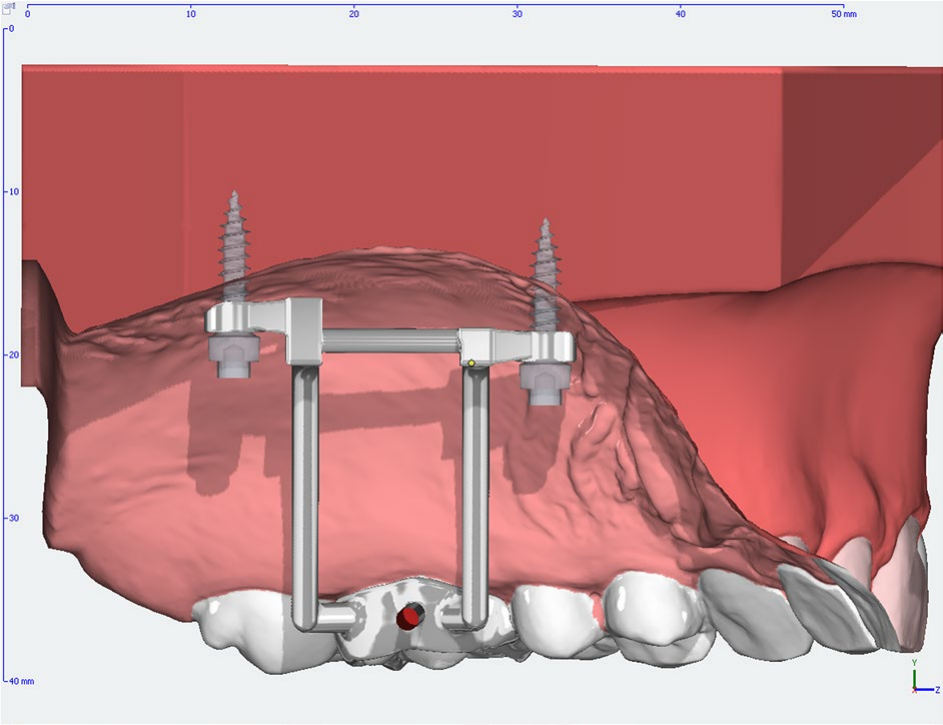
Miniscrew positioning with D-MED on an intra-oral scan

The Dutch Maxillary Expansion Device (D-MED) (Orthoproof, Nieuwegein, the Netherlands) is an individualized, 3D-designed, and fabricated appliance based on an intra-oral scan (IOS) (TRIOS 3 scanner, 3Shape, Copenhagen, Denmark). It is designed with OnyxCeph^3^™ (Image Instruments, Chemnitz, Germany) and 3D-printed with the Concept Laser Selective Laser Melting printer (CADdent, Augsburg, Germany). The stainless-steel structure (alloy: 60.5% cobalt, 28% chrome, 9% tungsten, and 1.5% silicon) includes two bands around the upper first molars and four rigid connectors with circular screw holes (internal diameter: 2.2 mm, external diameter: 3.6 mm, height: 1.8 mm). The connectors are designed to follow the curvature of the palatal shelves with a clearance of 3 mm, while the screw holes are located at a distance of 2 mm from the palatal mucosa, with the aim of avoiding mucosal overgrowth. Four self-tapping miniscrews (Quattro®, PSM Medical Solutions, Gunningen, Germany) connect the device to the palate through the screw holes. The position of the miniscrews is planned perpendicular to the occlusal plane, at 2 mm paramedian to the midpalatal suture in order to avoid nose septum perforation, anteriorly at the level of the upper second premolars and posteriorly at the level of the upper second molars, at a distance of ca. 2 mm from the junction of the hard and soft palate (see: Fig. 2). Following 3D-printing, the base of an expansion screw (Forestadent, Pforzheim, Germany) is soldered on the structure. The screw is positioned parallel to the palate at the level of the upper first molars and has an expansion capacity of either 10 mm or 12 mm, selected according to the required maxillary expansion.

### Clinical procedure

**Fig. 3:**
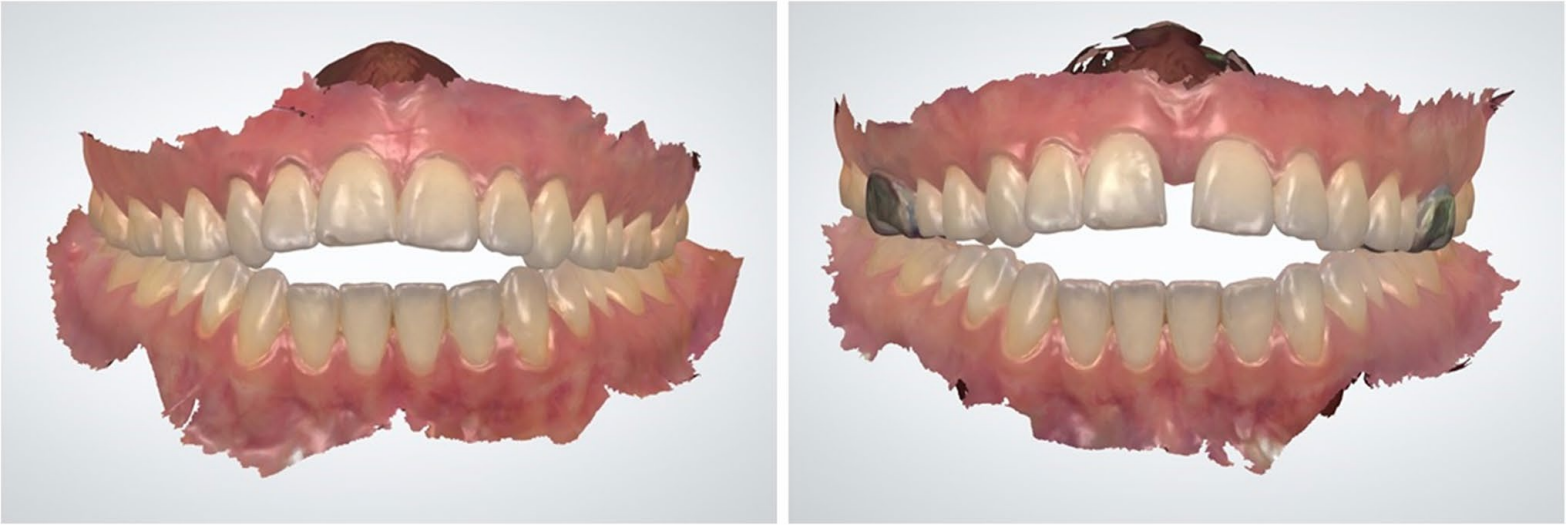
Frontal view of an intra-oral scan with D-MED at T0 (**A**: *left*) and T1 (**B**: *right*)

All procedures were performed by one orthodontist, the first author (A.K.), with the assistance of two senior orthodontists (J.S. and R.N.) in accordance with the clinical protocol for this MARPE device. As described above, an IOS was acquired for the design and fabrication of the D-MED (see: Fig. 3). Palatal thickness was measured on the pre-treatment CBCTs to determine the appropriate length of the miniscrews (11.0 mm or 13.0 mm; diameter: 2.0 mm) in order to achieve bicortical anchorage. When placing the D-MED, it was first cemented with light cure band cement (Ultra Band-Lok®, Reliance Orthodontic Products, Itasca, IL, USA) to facilitate the insertion of the miniscrews as designed, with the screw holes functioning as a surgical guide. Following local anaesthesia infiltration, an electric screw driver (iSD900, PSM Medical Solutions, Gunningen, Germany) with 40 Ncm torque at 20 rpm turning speed was used to insert the four miniscrews in the center of the screw holes, with the fitting diameter providing a tight grip. If the measured palatal thickness was > 6.0 mm, pre-drilling through the screw holes at the insertion sites of the miniscrews was performed.

**Fig. 4:**
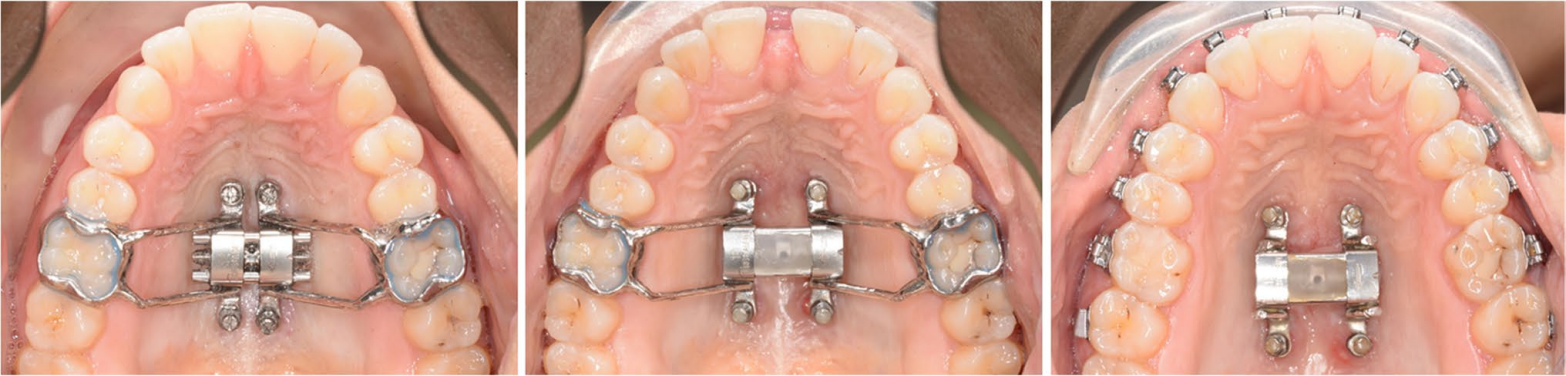
Maxillary occlusal view with D-MED at T0 (**A**: *left*), T1 (**B**: *middle*), and 3 months after expansion (**C**: *right*)

Maxillary expansion was initiated immediately after insertion of the D-MED (see: Fig. 4a). The screw was activated once a day, equivalent to 0.25 mm, and the expansion was closely monitored with weekly check-ups. The expansion was measured intra-orally with a digital calliper at the expansion screw, the central diastema, and inter-molar and inter-canine widths, and periodontal pockets deeper than 3 mm around the upper central incisors were recorded. The expansion was considered successful when the occlusal aspect of the palatal cusp of the upper first molars contacted the occlusal aspect of the buccal cusp of the lower first molars, and thus, when the necessary amount of expansion was achieved. The expansion screw was then blocked with resin-based composite. According to the retention protocol, the D-MED was left in place for 3 months after the end of expansion to allow remodelling of the bone in the separated midpalatal suture (see: Fig. 4b). After 3 months, the bands and connectors were removed and the treatment could be continued with a fixed straight-wire appliance, while the four miniscrews and the expansion screw were left in place for retention (see: Fig. 4c). The patients were then seen for a retention check-up at 6 and 12 months after termination of expansion. Finally, the expansion screw and miniscrews were removed either at 12 months or, in case of a surgical orthognathic treatment, prior to surgery.

### Data collection

IOS and CBCT scans were acquired before expansion (T0) and immediately at the end of expansion (T1). The CBCT scans were made on KaVo equipment (KaVo 3D eXam, KaVo Dental, Biberach, Germany) at 120 kV and 5 mA pulse mode and the patients were scanned seated upright, with the Frankfort horizontal plane parallel to the floor. At T0, the “Extended Height” modus was used (field of view: 23 cm diameter × 17 cm height; scan time: 17.8 s; voxel size: 0.3 mm) so it was also possible to extract an orthopantomogram and lateral cephalogram from the datafile for treatment planning purposes. At T1, the “Maxilla Only” modus was used (16 cm diameter × variable height; scan time: 14.7 s; voxel size: 0.2 mm) to reduce the radiation dose.

**Fig. 5:**
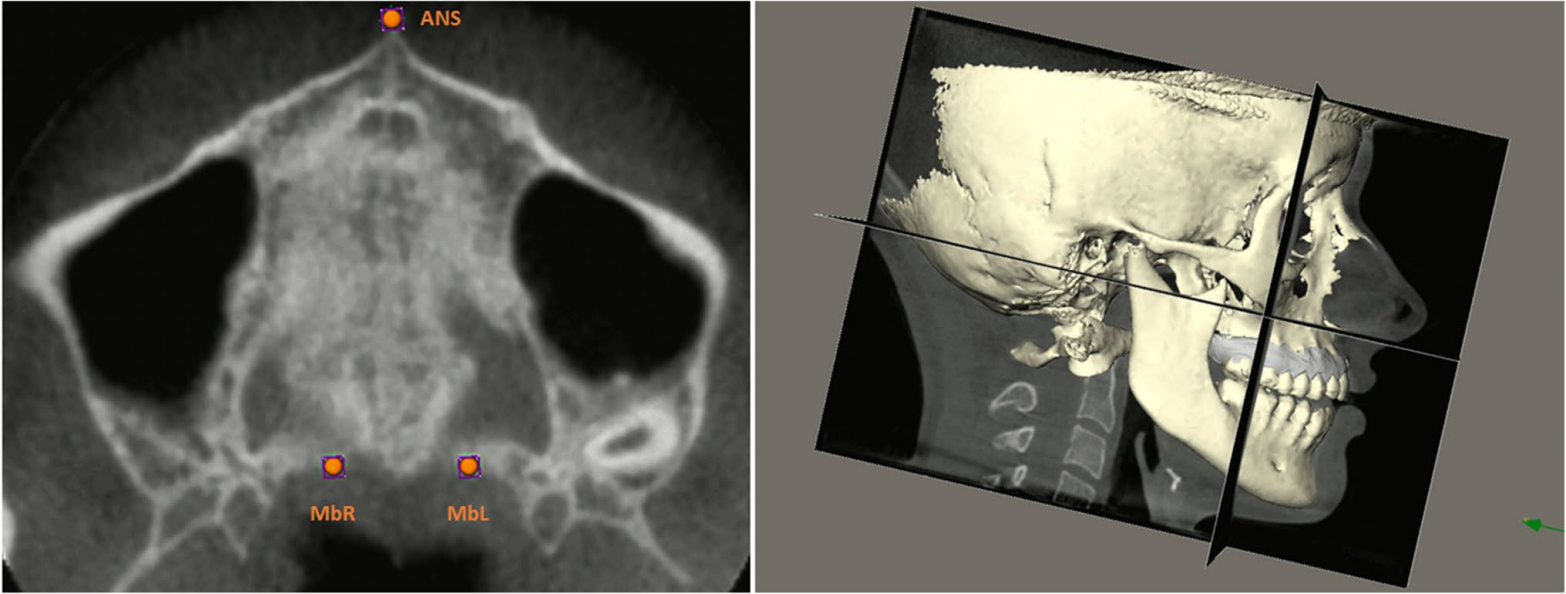
Orientation of the CBCT. (**A**: *left*) CBCT orientation of the horizontal plane defined by 3 landmarks: ANS-point, maxillary posterior border notch on the right (MbR), maxillary posterior border notch on the left (MbL). (**B**: *right*) CBCT orientation with 3D reference frame

The stereolithography files from the IOSs and the Digital Imaging and Communications in Medicine files from the CBCT scans were fused using IPS CaseDesigner® software (KLS Martin Group, Tuttlingen, Germany), creating separate fusion models for T0 and T1 per patient. The created models were imported into Maxilim® software (Medicim NV, Mechelen, Belgium) and a 3D reference frame was set up as follows: the horizontal plane was defined as a plane passing through the anterior nasal spine (ANS-point) anteriorly and the left and right notch located on the posterior maxillary border (see: Fig. 5a); the midsagittal plane was constructed as a plane perpendicular to the horizontal plane passing through the ANS-point and the middle of the line between the left and right posterior maxillary notch; the vertical (coronal) plane was constructed perpendicular to the horizontal and midsagittal planes (see: Fig. 5b). After setting up the reference frame, the dental and skeletal landmarks were identified manually on each fusion model.

### Measurements

**Fig. 6:**
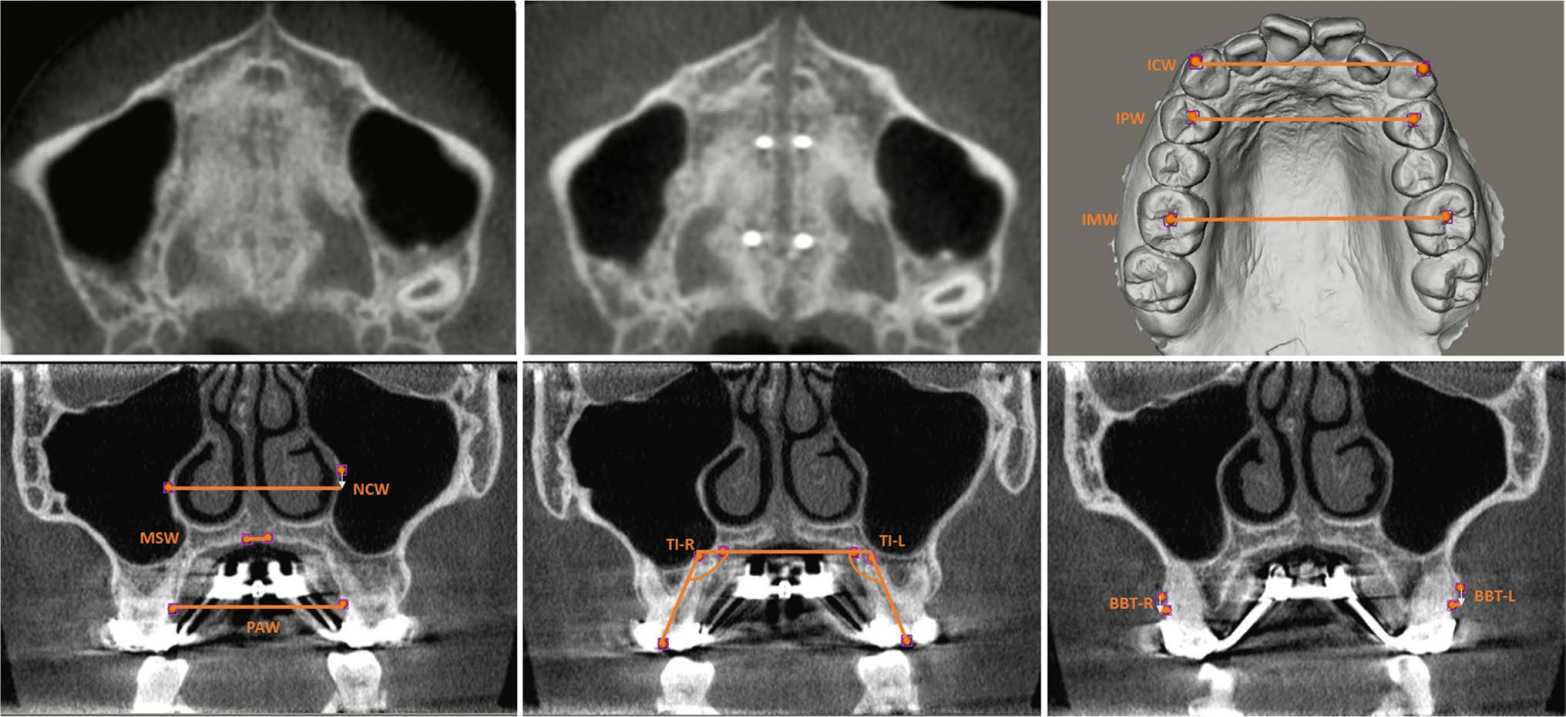
The hard palate at T0 and T1, and skeletal and dentoalveolar measurements on CBCT and IOS. (**A**: *upper left*) Hard palate at T0, before midpalatal suture separation, on an axial CBCT slice. (**B**: *upper middle*) Hard palate at T1, with separated midpalatal suture, on an axial CBCT slice. (**C**: *upper right*) Measurement of IMW, IPW, and ICW on an intra-oral scan. (**D**: *lower left*) Measurement of MSW, PAW, and NCW on a coronal cross-sectional CBCT slice through the buccal groove of the M1. (**E**: *lower middle*) Measurement of TI left and right on a coronal cross-sectional CBCT slice through the buccal groove of the M1. (**F**: *lower right*) Measurement of BBT left and right on a coronal cross-sectional CBCT slice through widest portion of the mesiobuccal root of the M1

The measurements were taken at T0 (see: Fig. 6a) and T1 (see: Fig. 6b). The transverse measurements were measured along the *X*-axis, while the vertical measurements were measured along the *Y*-axis. The primary outcome measure was the total transverse maxillary expansion at the level of the upper first molar and first premolar, defined as the change in intermolar width (IMW) or interpremolar width (IPW) between T1 and T0, respectively. The change in intercanine width (ICW) was measured as well (see: Fig. 6c). The total transverse maxillary expansion was the sum of skeletal expansion, alveolar expansion, and dental expansion. Skeletal expansion was defined as the midpalatal suture width (MSW) measured at T1. Alveolar expansion was defined as the change in palatal alveolar width (PAW) between T1 and T0, minus the MSW. Dental expansion was calculated by subtracting the skeletal expansion and the alveolar expansion from the total transverse maxillary expansion. The nasal cavity width (NCW) and the nasal cavity expansion (T1-T0) were also measured (see: Fig. 6d). The measurements were taken in the coronal plane on the CBCT at the level of the buccal groove of the first molar or the buccal cusp tip of the first premolar. Detailed descriptions of the measurements are shown in Table 1. The success rate of the treatment was the percentage of patients achieving the required maxillary width.

**Table 1:** Definitions of the measurements used in this study

Measurement	Section for measurements	Definition
Intermolar width (IMW)	3D reconstructed image from intra-oral dental scan	Transverse distance between the L & R maxillary M1 mesiocentral fossae
Interpremolar width (IPW)	3D reconstructed image from intra-oral dental scan	Transverse distance between the L & R maxillary P1 mesial fossae
Intercanine width (ICW)	3D reconstructed image from intra-oral dental scan	Transverse distance between the L & R maxillary canine (C) buccal cusp tips
Midpalatal suture width at M1 or P1 (MSW)	Coronal cross-sectional slice passing through the M1 buccal groove or the P1 buccal cusp tip	Transverse distance between the medial limits of the L & R palatine process at the palatal floor
Palatal alveolar width at M1 or P1 (PAW)	Coronal cross-sectional slice passing through the M1 buccal groove or the P1 buccal cusp tip	Transverse distance between the L & R most cervical medial limits of the palatal alveolar process
Nasal cavity width (NCW)	Coronal cross-sectional slice passing through the M1 buccal groove	Transverse distance between the L & R most lateral point of the nasal cavity
Tooth inclination at M1 or at P1 (TI)	Coronal cross-sectional slice passing through the M1 buccal groove or the P1 buccal cusp tip	2D inner angle between the palatal plane and the L & R line running through the palatal apex and the mesiobuccal fossa (M1) or the mesial fossa (P1)
Clinical crown height at M1 or P1 (CCH)	3D reconstructed image from Intra-oral dental scan	Vertical distance between the L & R most apical point of the gingival margin and the most caudal point on the buccal groove (M1) or buccal cusp tip (P1)
Buccal bone thickness at M1 or P1 (BBT)	Coronal cross-sectional slice passing through the most buccal point of the M1 mesiobuccal root or the P1 buccal cusp tip	Transverse distance between the L & R buccal cortex of the alveolar process and the mesiobuccal root (M1) or buccal root (P1)

The secondary outcome measures, assessing dental and periodontal side-effects, were tooth inclination (TI), as a measure of angular dental tipping (see: Fig. 6e), clinical crown height (CCH), as a measure of gingival recession, and buccal bone thickness (BBT), as a measure of buccal bone loss. These measurements were taken at T0 and T1 at the level of the first molar and first premolar on the left (L) and right (R) sides and mean values were used for statistical analysis. BBT at the first molar was measured at the mesiobuccal root because, given its greater proximity to the maxillary buccal alveolar cortex, it was found to be more prone to significant changes than the distobuccal root (see: Fig. 6f) [10]. Lastly, treatment duration of the expansion was recorded as the number of days from the first to the last day of activation of the MARPE appliance.

### Statistical analysis

A sample size analysis resulted in a minimum sample size of 33 individuals based on an alpha of 0.05, power of 95%, and an assumed effect size of 0.66 (G*Power, version 3.1.9.7., Dusseldorf, Germany). All measurements were performed by two independent observers (A.K. and B.O.). A random selection of 50% of the measurements was repeated by both observers individually after 3 weeks. Intra-observer reliability and inter-observer reliability were assessed using the intraclass correlation coefficient (ICC) and the duplicate measurement error (DME). The pre- and post-operative measurements were explored with descriptive statistics. Normal distribution of data was confirmed using the Kolmogorov–Smirnov test, except for mean buccal bone thickness at M1 at T1 and at P1 at T0. Paired *t*-tests, Mann–Whitney *U* test, and Wilcoxon signed ranks test were used to investigate changes between T0 and T1. The level of significance was set at 0.05. All statistical analyses were performed using SPSS® Statistics version 25.0 (IBM Corp., Armonk, NY, USA).

## Results

From October 2018 to September 2020, 35 patients were consecutively enrolled. One patient chose to terminate the orthodontic treatment after 2 days and this patient was excluded. Consequently, 34 patients (8 male, 26 female; mean age: 27.0 ± 9.4 years; age range: 17.1–56.0 years) were included for further analysis. The majority of patients were skeletally mature and presented with an Angle Class II/1 malocclusion with a posterior crossbite (see: Table 2). The maxillary expansion was successful in 32 patients (6 male, 26 female; mean age: 26.1 ± 8.2 years; age range: 17.1–47.5 years), resulting in a success rate of 94.1%. In two patients (2 men; 26.4 and 56.0 years old), separation of the midpalatal suture was not achieved as no diastema was observed clinically, in one due to a fracture of the expansion screw, and in the other due to substantial dental side-effects (buccal translation of the molars). The treatment time from initiation to termination of expansion with the MARPE appliance, the observation time, was on average 31.7 ± 8.0 days (range: 21–56 days). A post-hoc power analysis based on an alpha of 0.05, a sample size of 32, and an effect size of 3.86 (mean total expansion 6.56 mm and SD 1.7 mm) demonstrated that a power of 0.99 was obtained in this study.

**Table 2:** Basic characteristics of the total study population, as well as of the midpalatal suture separation and non-separation group

		Total	Separation	Non-separation
*N*		34	32	2
Age at start of expansion (years)	Mean ± SD	27.0 ± 9.4	26.1 ± 8.2	41.2 ± 20.9
	Range	17.1–56.0	17.1–47.5	26.4–56.0
Sex	Male	8	6	2
	Female	26	26	0
Angle classification	Class I	6	5	1
	Class II/1	20	20	0
	Class II/2	6	5	1
	Class III	2	2	0
Posterior occlusion	Unilateral crossbite	14	13	1
	Bilateral crossbite	4	4	0
	Anticipated crossbite	12	12	0
	Constriction without crossbite	4	3	1
Skeletal maturation stage	CVM4	3	3	0
	CVM5	12	12	0
	CVM6	19	17	2

*SD*, standard deviation; *CVM*, cervical vertebral maturation

### Inter- and intra-observer reliability analysis

Intra-observer reliability was excellent for both observer 1 (A.K.) (mean ICC: 0.94 ± 0.05; mean DME: 0.48 ± 0.38) and observer 2 (B.O.) (mean ICC: 0.92 ± 0.08; mean DME: 0.51 ± 0.35). Also, an excellent inter-observer reliability was found, with a mean ICC of 0.95 ± 0.06 and DME 0.43 ± 0.33.

### Maxillary expansion

The pre- and post-expansion intermolar, interpremolar, and intercanine widths are displayed in Table 3. The mean total transverse maxillary expansion at M1 and P1 was 6.56 ± 1.70 mm and 4.19 ± 1.29 mm, respectively (see: Table 4). The skeletal expansion (midpalatal suture width) at the level of M1 and P1 is shown in Tables 3 and 4. The mean skeletal expansion was 3.75 ± 1.02 mm at M1 and 3.86 ± 0.95 mm at P1. The mean anterior skeletal expansion was 3.0% (0.11 mm) larger than the posterior expansion (*p* = 0.29). After deducting the midpalatal suture width from the palatal alveolar width, the resulting value, defined as alveolar expansion or alveolar bone bending, was 0.68 ± 1.65 mm at M1 and 0.00 ± 1.22 mm at P1 (see: Table 5). The dental expansion, the component of expansion after deduction of the skeletal and alveolar expansion, was 2.12 ± 1.58 mm at M1 and 0.33 ± 1.16 mm at P1 (see: Table 5). All expansion results were statistically significant (*p* < 0.001). Based on these results, the contribution to expansion at M1 and P1 was 60.4 ± 20.1% and 92.2 ± 14.5% skeletal, 8.1 ± 27.6% and 0.0 ± 18.6% alveolar, and 31.6 ± 20.1% and 7.8 ± 17.7% dental, respectively (see: Table 5).

**Table 3:** Mean (± SD) dental and skeletal measurements (mm) at M1, P1, and/or C at T0 and T1

	Interdental width		Midpalatal suture width		Palatal alveolar width		Nasal cavity width	
Position	T0	T1	T0	T1	T0	T1	T0	T1
M1	43.50 ± 3.08	50.06 ± 3.08	0	3.75 ± 1.02	30.96 ± 2.67	35.39 ± 3.05	31.95 ± 2.53	34.02 ± 2.94
P1	32.59 ± 2.82	36.78 ± 3.20	0	3.86 ± 0.95	25.19 ± 1.96	29.05 ± 2.58		
C	32.53 ± 2.79	36.72 ± 3.25

**Table 4:** Mean (± SD) skeletal and dentoalveolar expansion (mm) (T1-T0) at M1, P1, and/or C

Position	Total expansion	Midpalatal suture separation	Alveolar width increase	Nasal cavity expansion
M1	6.56 ± 1.70***	3.75 ± 1.02***	4.43 ± 1.60***	2.07 ± 1.42***
P1	4.19 ± 1.29***	3.86 ± 0.95***	3.86 ± 1.49***	
C	4.19 ± 1.57***			

Paired *t*-tests were performed; ***p < 0.001

**Table 5:** Mean (± SD) skeletal, alveolar, and dental component (mm; %) of total expansion at M1 and P1

Position	Skeletal expansion	Alveolar expansion	Dental expansion
M1	3.75 ± 1.02 mm 60.4 ± 20.1%	0.68 ± 1.65 mm 8.1 ± 27.6%	2.12 ± 1.58 mm 31.6 ± 20.1%
P1	3.86 ± 0.95 mm 92.2 ± 14.5%	0.00 ± 1.22 mm 0.0 ± 18.6%	0.33 ± 1.16 mm 7.8 ± 17.7%

The mean nasal cavity widths at T0 and T1 at the level of M1 are shown in Table 3. The nasal cavity expansion, the mean difference between T1 and T0, was 2.07 ± 1.42 mm (*p* < 0.001; see: Table 4), which was 55.2% of the skeletal expansion.

### Side-effects of expansion

The mean values of side-effects at T0 and T1 and their difference (T1-T0) are shown in Table 6. The mean increase in tooth inclination was 3.88 ± 3.92° (*p* < 0.001) at M1 and 2.29 ± 3.89° (*p* < 0.01) at P1. Clinical crown height increased on average 0.12 ± 0.31 mm (*p* < 0.05) at M1 and 0.04 ± 0.22 mm (*p* = 0.31) at P1. Buccal bone thickness decreased on average 0.31 ± 0.49 mm at M1 (*p* < 0.01) and 0.01 ± 0.45 mm at P1 (*p* = 0.91).

**Table 6:** Mean (± SD) TI (degrees), CCH (mm), and BBT (mm) at T0, T1, and T1-T0 at M1 and P1

	Tooth inclination			Clinical crown height			Buccal bone thickness		
Position	T0	T1	T1-T0	T0	T1	T1-T0	T0	T1	T1-T0
M1	116.60 ± 6.32	120.48 ± 6.58	3.88 ± 3.92***	5.69 ± 0.84	5.81 ± 0.87	0.12 ± 0.31*	1.35 ± 0.69	1.04 ± 0.62	− 0.31 ± 0.49***
P1	106.67 ± 6.28	108.96 ± 6.15	2.29 ± 3.89***	7.51 ± 0.84	7.55 ± 0.89	0.04 ± 0.22	1.16 ± 0.67	1.15 ± 0.63	− 0.01 ± 0.45

Wilcoxon signed ranks tests were performed; * *p* < 0.05; *** *p* < 0.001

## Discussion

### Study and appliance design

The aim of the present study to provide a higher degree of evidence on the efficacy of of MARPE was supported by a number of methodological choices. In contrast to most previous studies, a prospective study design was applied. The selection bias, one of the main shortcomings of previous reports [8], was reduced to a minimum through the inclusion of consecutive patients who presented with transverse maxillary discrepancy. Regarding the minimum age for inclusion, a clinical approach was followed, as the age of 16 is the clinically applied cut-off point between RPE and SARPE. Furthermore, a sufficient population size was present to provide the power that was required.

The D-MED, developed in 2018, was the first 3D digitally designed, patient-specific, and 3D-printed MARPE appliance with the aim of delivering more predictable expansion results. The 3D-printed steel structure aimed to provide more rigidity, decreasing the risk of fracture. The accompanying miniscrews (2.0 mm diameter) were thicker than the miniscrews used in other MARPEs (1.5 mm [11, 12] or 1.8 mm diameter [13–18]), delivering a stronger anchorage and transmission of expansion forces to the palate. Based on the height of the screw holes, the distance to the palatal mucosa, the thickness of the mucosa, and of the palate, 11 mm or 13 mm miniscrews were applied to ensure bicortical anchorage in all cases, even though for some patients with very thin palatal bone, shorter miniscrews could have been appropriate as well.

### Efficacy of MARPE

The mean age (27.0 ± 9.4 years) of the study population was significantly higher than in previous MARPE studies (range: 20.1 ± 2.4–24.9 ± 1.8 years) [11–23]. Even with the higher mean age, the success rate of MARPE by D-MED is comparable to and even somewhat higher than the mean success rate of MARPE (92.5%) [8]. One of the two patients whose midpalatal suture did not separate was significantly older (56.0 years), which is in line with the findings of Shin et al. and Jeon et al. that there is a significant negative correlation between age and midpalatal suture opening by MARPE [18, 23].

The total transverse expansion (IMW) by D-MED was comparable to the mean by MARPE (6.55 ± 1.05 mm) [8] and by SARPE (7.0 ± 0.85 mm) [24]. However, the amount of skeletal expansion by D-MED was significantly larger than the mean by MARPE (2.33 ± 0.70 mm) [8] and larger than that by SARPE (3.3 ± 0.55 mm) [24]. Concordantly, the percentage of skeletal expansion by D-MED (60.4%) was significantly higher than by MARPE (35.6%) [8] and by RPE and SARPE, ranging from 40 to 55% [25, 26] and 21.5 to 46.3% [27–30], respectively. Considering the differences in amount of required expansion, the extent of screw activation differed between patients, leading to a greater variance in the absolute measured values. Standard deviations of the contribution of alveolar, skeletal, and dental expansion were calculated to express this variance. In comparison to the M1, the expansion at P1 was almost fully skeletal. This could be attributed to the expansion forces being directed at the first molars via the bands and the four rigid connectors, which led to more dentoalveolar expansion at M1, while this was nearly absent at P1 level. There was also great variance in the age of the participants. A recent study by Jia et al. on the age-dependent effects of MARPE found a significant difference in the amount of skeletal expansion between patients younger and older than the age of 20 [31]. In the current study, patients < 20 years (*N* = 8) had a skeletal component of 72.1% and patients ≥ 20 years (*N* = 24) of 56.5% (*p* = 0.056), demonstrating a similar age-related effect. The expansion was achieved in a short time span of ca. 31 days, comparable to other MARPE studies with a rapid expansion protocol (range: 27–35 days) [11, 15, 17].

There was no statistically nor clinically significant difference in suture width at M1 and P1, showing a parallel anteroposterior midpalatal suture opening pattern, as was also found in previous studies [16, 32]. On the other hand, the expansion at the nasal cavity indicated that MARPE affected not only the maxilla, but also the circummaxillary structures with a more pyramidal or V-shaped expansion pattern in the coronal plane. These findings coincide with previous reports on RPE [33, 34], SARPE [29, 30], and MARPE [17, 31, 32], while a few MARPE studies reported a parallel expansion pattern [6, 16].

Considering that there are no major differences in expansion protocol between the available MARPE appliances, the favorable skeletal expansion results in this study could be attributed to the individualized design and larger diameter of the miniscrews, which led to better fitting and increased anchorage and force transmission with the D-MED. A precise comparison of the MARPE appliances, however, was complicated due to methodological differences of the studies.

### Side-effects of MARPE

As described in previous RPE [10, 35, 36], MARPE [14–17], and SARPE [2, 3, 27] studies, expansion with the D-MED in the present study also had some side-effects. Buccal crown tipping was potentially clinically significant, as the consequential buccal alveolar crest height decrease could lead to gingival recessions [17]. Different factors, such as tipping of the miniscrews, low palatal thickness, or bone density, could have contributed to tipping, as they could have led to increased force delivery on the anchoring molars [18, 37]. However, the mean increase of clinical crown height was not clinically relevant, as well as the decrease of buccal bone thickness, which was similar to that found in earlier MARPE reports [15–17], while studies on RPE presented contrasting results, with minimal changes in some [35], and a significant decrease in others [10, 36]. However, in patients with a weak periodontium, thinning of the buccal bone could be a potentially relevant side-effect, as these patients may be more prone to further periodontal deterioration [10]. This was the case in one out of two failed expansions observed in this study.

### Study limitations and future perspectives

Considering study design, ideally, an RCT would provide stronger scientific evidence by comparing the effects of MARPE and SARPE. However, due to the surgical nature of SARPE, random allocation of patients to a surgery and a non-surgery group may face challenging ethical issues. In order to address concerns pertinent to the ALARA principle of radiation exposure, the CBCT at T1 had a smaller field of view. On the other hand, this created a challenge relevant to consistency between the first and the second image acquisition, which, nonetheless, was overcome by the selection of the reference frame.

Despite the positive treatment outcome of the D-MED in the immediate post-expansion period, the observation time was very short and studies regarding the long-term effects and stability following MARPE expansion would be recommended. Due to the limited observation time, it was not possible to evaluate root resorption as a potential side-effect. Furthermore, as there were only two failures, a reliable prediction on which participants might not achieve a successful expansion could not be made. This indicates the need for further research on the prognostic factors, such as palatal morphology in a prospective clinical setting. Nonetheless, it can be expected that the use of MARPE for the correction of transverse maxillary discrepancy will continue to increase.

## Conclusions

This prospective clinical cohort study contributes to a higher degree of evidence on the efficacy of MARPE, which achieved a significant transverse maxillary expansion in a large and diverse group of late adolescents and adults.

The Dutch Maxillary Expansion Device demonstrated to be an effective MARPE appliance with a success rate of 94.1%. Moreover, the skeletal component of expansion was very high both at molar and at premolar levels, 60.4% and 92.2%, respectively. Buccal dental tipping, clinical crown height increase, and buccal bone thinning were observed, but could be considered clinically insignificant in patients with a healthy periodontium.

Therefore, MARPE is an effective and safe non-surgical treatment for transverse maxillary deficiency in late adolescents and adults.
